# Supplementary material for: Association between oral microbiome and seven types of cancers in East Asian population: a two-sample Mendelian randomization analysis
Source: Front Mol Biosci. 2023 Nov 21;10:1327893. doi: 10.3389/fmolb.2023.1327893 (PMC10702768; doi:10.3389/fmolb.2023.1327893)
Supplement: Supplementary file 1 [file DataSheet1.ZIP › Supplementary Materials/MR plots for tongue/tongue═╝/Pancreatic cancer/╥╚╧┘░⌐_╔╕╤í╜ß╣√/pheno.486_to_pancreatic cancer_forest.pdf]

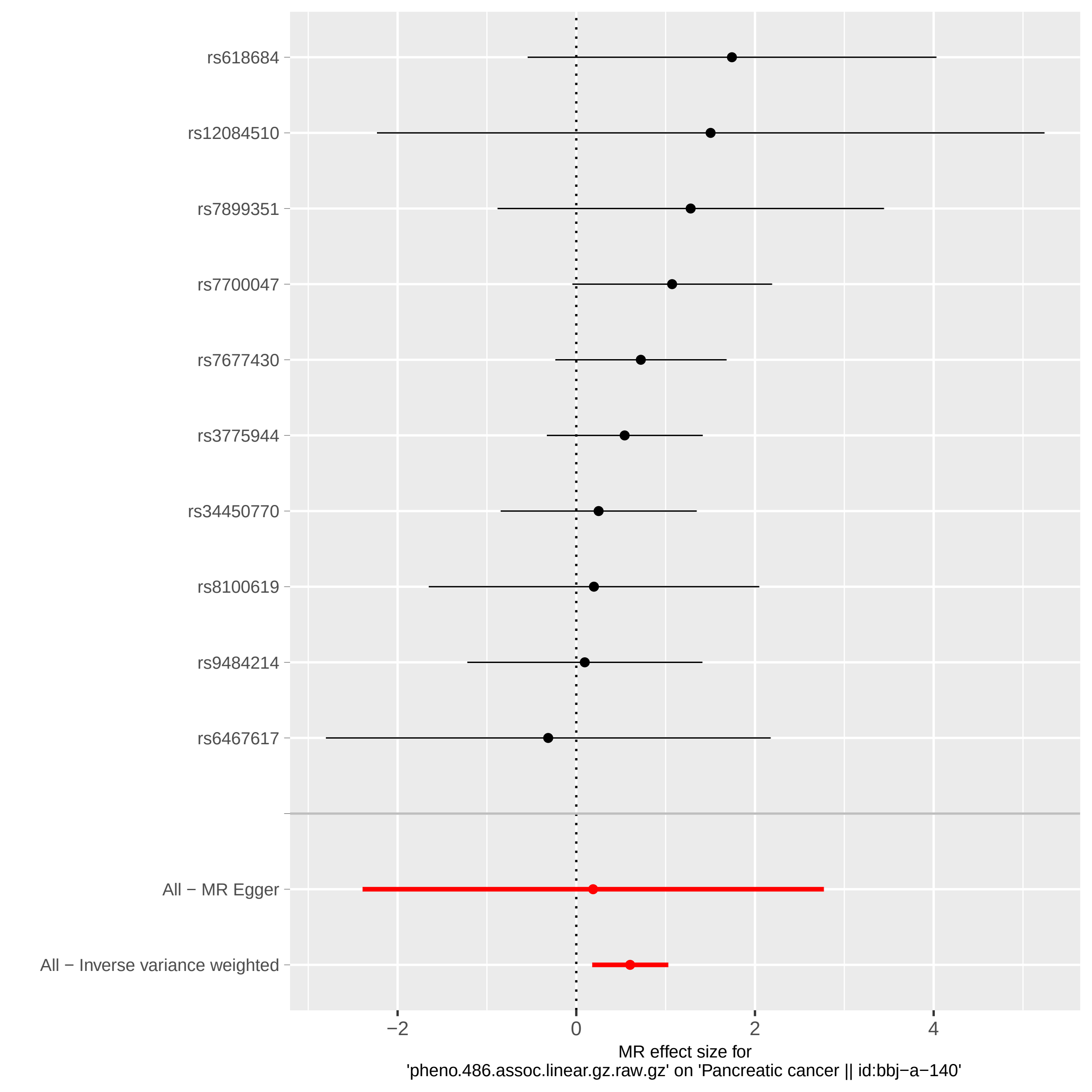
rs618684
rs12084510
rs7899351
rs7700047
rs7677430
rs3775944
rs34450770
rs8100619
rs9484214
rs6467617
All − MR Egger
All − Inverse variance weighted
−2
0
2
4
MR effect size for
'pheno.486.assoc.linear.gz.raw.gz' on 'Pancreatic cancer || id:bbj−a−140'
